# Seroprevalence and socioeconomic impact of the first SARS-CoV-2 infection wave in a small town in Navarre, Spain

**DOI:** 10.1038/s41598-023-30542-x

**Published:** 2023-03-08

**Authors:** Marta Ribes, Júlia Montañà, Marta Vidal, Ruth Aguilar, Patricia Nicolás, Uxue Alfonso, Natalia Rodrigo, Carlo Carolis, Carlota Dobaño, Gemma Moncunill, Carlos Chaccour

**Affiliations:** 1 ISGlobal, Hospital Clínic, Universitat de Barcelona, Barcelona, Spain; 2 Centro de Investigação em Saúde de Manhiça, Manhiça, Mozambique; 3 IMPACT Initiatives, Geneva, Switzerland; 4 Centre for Genomic Regulation (CRG), The Barcelona Institute of Science and Technology, Barcelona, Spain; 5 CIBERINFEC, Madrid, Spain; 6 Universidad de Navarra, Pamplona, Spain

**Keywords:** Infectious diseases, Infectious diseases, Immunology

## Abstract

The characterization of the antibody response to SARS-CoV-2 and its determinants are key for the understanding of COVID-19. The identification of vulnerable populations to the infection and to its socioeconomic impact is indispensable for inclusive policies. We conducted an age-stratified cross-sectional community-based seroprevalence survey between June 12th and 19th 2020—during the easing of lockdown—in Cizur, Spain. We quantified IgG, IgM and IgA levels against SARS-CoV-2 spike and its receptor-binding domain in a sample of 728 randomly selected, voluntarily registered inhabitants. We estimated a 7.9% seroprevalence in the general population, with the lowest seroprevalence among children under ten (n = 3/142, 2.1%) and the highest among adolescents (11–20 years old, n = 18/159, 11.3%). We found a heterogeneous immune-response profile across participants regarding isotype/antigen-specific seropositivity, although levels generally correlated. Those with technical education level were the most financially affected. Fifty-five percent had visited a supermarket and 43% a sanitary centre since mid-February 2020. When comparing by gender, men had left the household more frequently. In conclusion, few days after strict lockdown, the burden of SARS-CoV-2 infection was the lowest in children under 10. The findings also suggest that a wider isotype-antigen panel confers higher sensitivity. Finally, the economic impact biases should be considered when designing public health measures.

## Introduction

The COVID-19 pandemic has caused—as of July 21st 2022—564,126,546 confirmed cases and 6,371,354 confirmed deaths worldwide^1^, although the actual number of deaths has been estimated to be three times higher^2^. Vaccination has resulted in a decrease in mortality and the number of severe COVID-19 cases, and in a relaxation of non-pharmacological public health preventive measures. However, the lack of vaccines preventing infection, the inequitable access to them^3^, and the appearance of immune-evasive viral variants with higher transmission capacity has led to sustained viral transmission in many settings. Seroprevalence studies are essential to estimate the burden of infection and identify vulnerable populations, but also to characterize the antibody response, critical for surveillance and diagnostics.

In Spain, the cumulative effects of an initial prolonged lockdown and high burden of disease particularly hit an economy that relies heavily on tourism and the service sector. On the 31st of January the first COVID-19 confirmed case in Spain was diagnosed in a tourist in La Gomera. On the 25th of February, the first three locally transmitted cases were detected in Madrid, Barcelona and Castelló. The first case reported in Navarre dated from the 28th of February^4^. Lockdown in Spain started on the 14th of March 2020 with the only exception of acquiring essential goods, receiving healthcare, returning to the usual place of residence or not having the possibility of teleworking. From the 26th of April, children under 14 were allowed to play for one hour in the street. In Navarre, from the 11th of May, people were allowed to gather in groups of 10 in open spaces and terraces, practice sports in open spaces and without physical contact, visit museums, shops and theaters and places of worship. From the 25th of May people were allowed to gather in groups of 15, and in groups of 20 in outdoor activities, and restaurants reopened indoor spaces with capacity restrictions. The 21st June normality was restored with some public health measures remaining such as the use of face mask in public spaces and the promotion of telework^5,6^. Estimates show a cumulative increase of 11.9% in the rate of unemployment in the service sector during 2020. In the Autonomous Community of Navarre, this proportion was estimated at 10%. Remarkably, no specific association has been found between COVID-19 incidence and economic downturn measured as reduction in gross domestic product^7^. Bárcena-Martín and Cantó identified youngest individuals to be the most vulnerable to the impact of the economic crisis, whereas individuals with a higher educational level had greater income stability during the crisis caused by the pandemic^8^.

During the first epidemic wave from March until June 2020, quantitative real-time reverse transcription polymerase chain reaction (RT-qPCR) testing in Spain was restricted to severe patients and/or high-risk groups (mainly health care workers), leading to many undetected cases^4^. Other factors limited the representability of reported cases: some individuals might not seek medical care if symptoms were mild, variable testing availability depending on the setting, or incomplete case reporting to public health authorities. In the absence of widespread RT-qPCR or rapid diagnostic tests (RDT), serologic assays—by which antibodies against the virus are measured—allowed a more comprehensive identification of those individuals who had been formerly infected, including those who experienced mild disease or sub-clinical infections. Former infections identified from serologic assays are thus not biased by health care–seeking behavior or testing availability^9^. This information was essential to estimate the true proportion of the population that had been infected by the virus and thus improve disease burden estimates, know the proportion of asymptomatic or mildly symptomatic cases, and inform policy decisions related to restrictive measures. In Navarre, up to the 28th of June, 10,358 cases had been declared (1.57% of the population)^4^. In Spain, the ENE-COVID, a nationwide seroprevalence survey study, estimated a seroprevalence of 5.2% (95% CI 4.9–5.5%) nationwide between the 8th and 20th June 2020, and of 6.6% (95% CI 5.1–8.5%) in Navarre. No differences were found between sex, and the lowest prevalence was found in babies and children younger than 10 years^10^.

A robust body of evidence has confirmed that the vast majority of SARS-CoV-2 infected patients develop an antibody response, which is stronger in symptomatic and severe COVID-19 cases^11^. Immunoglobulins (Ig) type M, G and A peak around 20–30 days post symptoms-onset, after which IgM levels start to decrease while IgG levels—and to a lesser extent IgA—are more persistent^12,13^. IgGs have been detected up to 16 months after infection^14^. Although this long maintenance of IgGs sheds light in the end of the pandemic, the threshold of antibody levels conferring protection against infection or severe disease is still not known. Despite antibodies protecting against infection to a certain degree^12^, re-infections are recurrent but—comfortingly—risk of disease severity is significantly decreased after infection or vaccination^13^.

Here we present a seroprevalence study in a town of 3,925 inhabitants in Spain conducted in June 2020. Three isotypes (IgG, IgM and IgA) were measured against two SARS-CoV-2 antigens (the spike [S] and its Receptor Binding Domain [RBD]) in a random subsample drawn from voluntarily registered inhabitants.

## Methods

### Study design, study area and selection of participants

This study was a cross-sectional age-stratified community-based seroprevalence survey that took place between the 12th and the 19th of June 2020 based on a voluntary response sample. The study area was the Cendea de Cizur, a Spanish municipality in the Autonomous Community of Navarre, located at 7 km from the capital of the autonomous community, Pamplona, and belonging to its metropolitan area. The municipality has a surface of 46.5 km^2^ and had 3,925 inhabitants as of April 2020^15^. It is divided in eight councils: Astráin, Cizur Menor—which is the most populated—, Gazólaz, Larraya, Muru-Astráin, Paternáin, Undiano and Zariquiegui. Due to its proximity to the capital of the region, many inhabitants commute regularly for work, educational or recreational purposes. The town has the highest gross salary and one of the lowest risks of poverty and Gini index (9.23% and 24.7% respectively) in Navarre^16^.

Due to the impossibility of accessing personal data of the inhabitants of the town, the sampling frame used was a voluntary registry of the municipality inhabitants. The study was announced on the town hall official accounts on social media and was sent via mail post to all residents. To participate, they had to register filling out an online form, by phone or in paper in the town hall where their contact information was gathered. Using the municipal register as sampling frame, participants were then selected by simple randomization stratified by age group in five age strata. Weighted sampling was done according to the following five age groups: 0–10, 11–20, 21–50, 51–65, > 65 years old.

The inclusion criterion was being a resident of the municipality of Cendea de Cizur who signed-up for the survey. Exclusion criteria were: (i) being absent from the household at the time of the visit, (ii) not answering the phone after five attempts in at least two different days at different daytimes. Assuming a 5% prevalence of SARS-CoV-2 seropositivity at the time of the survey, a total sample of 775 participants was aimed to estimate the seroprevalence within a 3% margin of error and 95% confidence.

### Household visit procedures

Out of the total volunteers registered, a random sample was selected according to the size needed per strata. Randomly selected individuals from this list were approached telephonically to schedule a household appointment and enrolled upon review of eligibility criteria. When an individual was not eligible for recruitment, a substitute was retrieved randomly without replacement from the rest of volunteers in the register.

Informed consent was obtained from each patient before recruitment after explaining the study's objectives. In the case of minors, parents had to provide consent for them, all adolescents between 12 and 17 years of age were further required to sign an informed assent.

Afterwards, a brief epidemiological questionnaire was administered to all survey participants. This covered the following information: participant´s demographic characteristics, targeted medical history, current and past health status (with special emphasis on possible symptoms and signs of COVID-19), exposure to COVID-19 cases or contacts of cases (confirmed or not), other possible sources of infection, socioeconomic status and COVID-19 impact on it, and prevention measures taken in the context of the COVID-19 epidemic. When asked for COVID-19 related-symptoms and behaviours, the period between mid-February and the interview day was considered since the first COVID-19 locally transmitted confirmed case in Spain dated from the 25th of February. Data were collected using standardized electronic questionnaires through smartphones and tablets.

The fieldworker collected a capillary blood sample in an EDTA tube via finger-prick for the determination of antibodies. Samples, and laboratory registers were identified with a unique participant identification number.

### Laboratory procedures

Blood samples were transported from the field to the biobank in the University of Navarra by cold chain using coolers. At the end of each day, blood was centrifuged, and the plasma separated and kept at -80ºC in the laboratory until the shipment to ISGlobal for serological analysis.

We measured the levels of three antibody isotypes (IgG, IgM and IgA) against the SARS-CoV-2 S glycoprotein, produced at the Centre de Regulació Genòmica (CRG), and RBD, kindly donated by the Krammer lab (Mount Sinai, New York)^17^ using a previously validated method based on quantitative suspension array technology (xMAP®, Luminex®)^18^. This method, using IgG, IgM and IgA isotypes and RBD and S antigens yielded a sensitivity of 83% and a specificity of 95%^19^.

Plasma samples were incubated with MagPlex® Microspheres coated with the S and RBD antigens. After wash, beads were incubated with anti-human Ig labelled with fluorescent phycoerythrin and resuspended with an assay buffer and read in a Luminex® 100/200 equipment for quantification of bound IgG, IgM and IgA. Levels of antibodies were expressed in median fluorescence intensity (MFI). Seropositivity was determined based on a threshold calculated as 10 to the mean plus 3 standard deviations of log_10_-transformed MFIs of 71 negative controls (pre-pandemic samples from adults ranging 20–60 years of age). All methods were carried out in accordance with relevant guidelines and regulations^20^.

At the time of protocol development, the S antigen from the Wuhan strain was chosen because it was the leading vaccine candidate target and one of the most immunogenic. RBD—the fragment of S protein that mediates binding of the virus to the host receptor ACE2 in the lung cells—was also analyzed because IgG levels to RBD correlated with the levels of neutralizing antibodies that had been associated with protection^21^.

### Statistical analysis

Seroprevalence of SARS-CoV-2 was estimated overall and stratified by isotype and age group. The seroprevalence for the whole population was estimated considering the weights of the sampling per age group. Seroprevalence estimates were also corrected for the finite population correction factor, used when sampling without replacement from more than 5% of a finite population. Although the study did not use household cluster sampling, the target population was small and the sample included 25% of the entire population that was large enough to include multiple members of the same household. This makes the variance inter-participants unequal due to probable household clustering. To account for this, we used robust variance estimates.

Study participants’ characteristics were described via mean and standard deviation (quantitative variables) and percentages (categorical variables). Chi-square or Fisher’s exact test (for categorical variables) and T-test (for continuous quantitative variables) were used to test the association between certain variables of interest.

Venn Diagrams were created to illustrate the overlap between antigens and between the three isotypes. We assessed the correlations between log_10_-transformed MFIs with Spearman’s rank test. Locally estimated scatterplot smoothing (LOESS) was used to visualize the non-parametric correlation.

Univariable and multivariable logistic regression models were run to evaluate factors associated with being seropositive and with being seropositive specifically for each isotype among the seropositive overall. Variables that had a *p*-value < 0.2 in the univariable analysis were included in the multivariable models. Categorical variables with less than six observations in one of the categories were discarded as well as observations with missing data. Explanatory variables included were sex, age, town, body mass index (BMI), blood group, Bacillus Calmette–Guérin (BCG) vaccination, flu vaccination, having had flu or a cold in last winter season, having any comorbidity, allergy, reporting COVID-19 compatible symptoms since mid-February 2020, having had a close COVID-19 contact, COVID-19 diagnosis, number of members in the household, highest schooling level and exposure to gatherings. A stepwise selection model by AIC was used. Beta coefficients were transformed to Odds Ratios.

Univariable and multivariable linear regression models were run among the seropositive participants to evaluate factors associated with isotype-specific antibody levels against SARS-CoV-2. Variables that had a *p*-value < 0.2 in the univariable analysis for any of the antigens (RBD, S or their sum) were included in the isotype-specific multivariable model. The days post symptoms onset (pso) were also included to account for time passed since infection. Categorical variables with less than six observations in any of the categories were discarded as well as observations with missing data. Explanatory variables included were sex, age, BMI, days post symptom onset, body ache/fatigue, upper respiratory symptoms, allergy, fever, smoker, anosmia/ageusia, lower respiratory symptoms, gastrointestinal symptoms, having had a cold in last winter season. Beta coefficients were transformed to percentages to be more easily interpreted.

A *p*-value of ≤ 0.05 was considered statistically significant and 95% CIs were calculated for all estimates. We performed the statistical analysis with Stata v14.2 (College Station, TX:StataCorp LLC) and with R version 4.0.3 (packages tidyverse^22^, ggplot2^23^, st^24^, sjPlot^25^).

The protocol was reviewed and approved by the ethics committee Comité de Ética de la Investigación con medicamentos (PI_2020/54).

## Results

### Characteristics of the study participants

**Figure 1:**
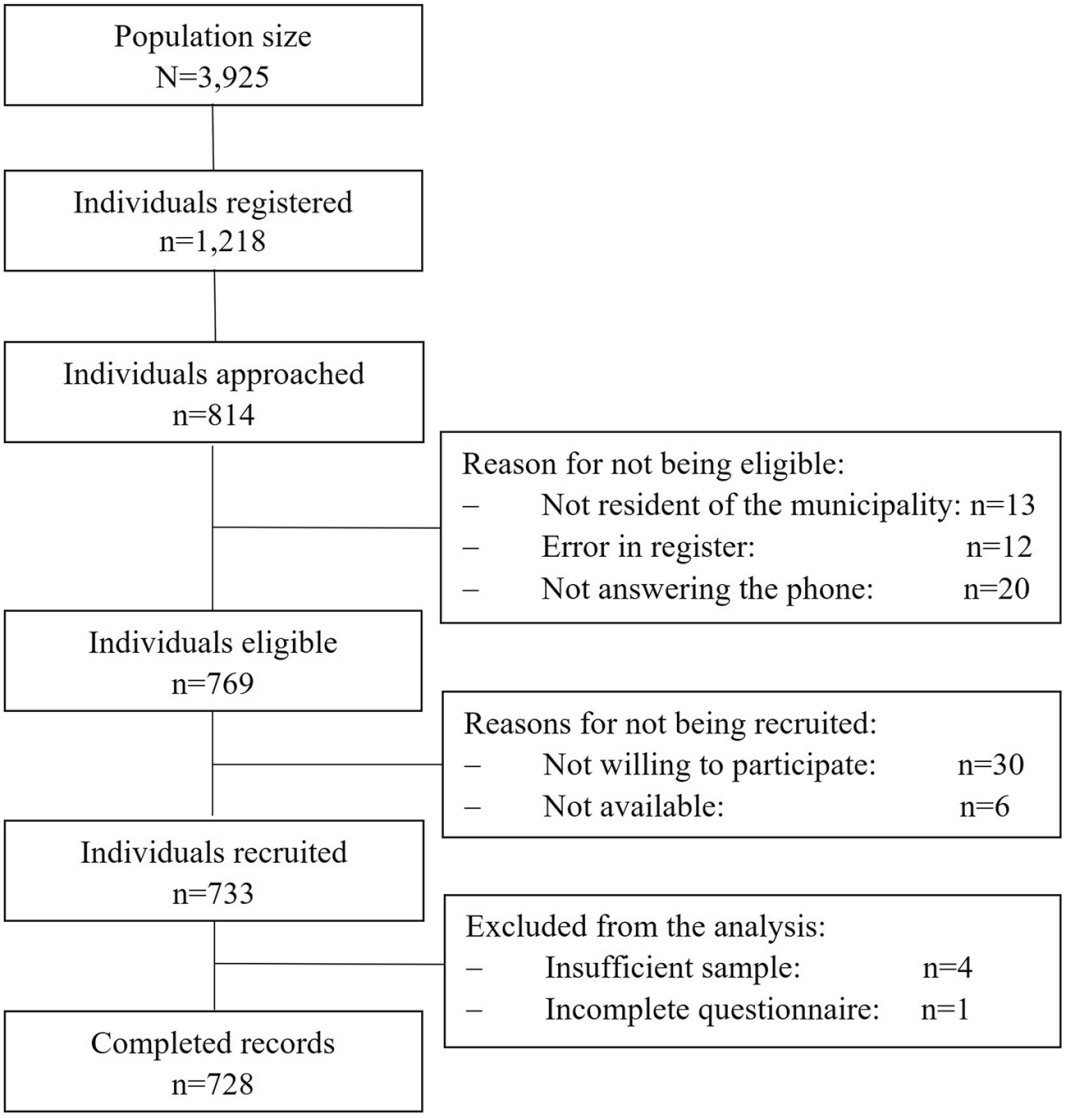
Study participants’ flowchart.

Out of the total population censed in Cendea de Cizur, 1,218 inhabitants voluntarily registered for the study. Altogether, 814 randomly selected individuals were approached by phone call, of which 769 were eligible. Among the reasons for not being eligible: (i) 13 individuals approached were not residents of the municipality of Cendea de Cizur, (ii) in 12 an error had been made in the register and the individual was unlocalizable, (iii) 20 did not answer the phone after a minimum of five phone calls in at least two different days and different times of the day. Out of the 769 individuals who were eligible, 733 were recruited, yielding a participation rate of 95.3%, which was expected as individuals had already expressed their willingness to participate. Five participants were further excluded from the analysis because of insufficient sample collected or because of incomplete questionnaire (Fig. 1).

Sufficient participants were recruited for each age strata except for the group over 65 years of age, since only 97 people older than 65 registered for the study, of which 93 were recruited while the sample size needed was 130 (Supplementary Table 1). There was a large overlap between the age distribution of the population and the sample obtained.

Half of participants were female (365/728, 50.1%) (Table 1 and Supplementary Table 2). Thirty-three percent across all ages had had flu symptoms or a cold during last transmission season up to mid-February (the week with the highest number of flu diagnosis was the 4th of January). Twenty percent had received the flu vaccine, 65.6% in those over 65 years (in Navarre these figures were 18.6% and 61% respectively)^4^. Forty-six percent declared having comorbidities, some known to be risks factors for severe COVID-19 disease (Supplementary Table 10). Regarding COVID-19 related characteristics, 54% of participants declared having had any compatible symptoms (fever, chills, fatigue, myalgia, sore throat, anosmia, ageusia, cough, rhinorrhea, dyspnea, wheezing, chest pain, headache, abdominal pain, diarrhea, sputum) from mid-February 2020 until date of interview (mid-June 2020); and 33.6% had had a close contact with a suspected or confirmed case of COVID-19. Ten participants had been previously diagnosed with COVID-19, seven of them only by clinical evaluation, one via RT-qPCR and two via a serology test. Seventy-seven percent had been to a group gathering by having visited a health center, grocery shop, bank, church, hairdresser, or another city from mid-February until the end of March 2020.

**Table 1:** Characteristics of study participants.

Characteristics	Total (n = 728)
Sex^a^	
Male	363(49.9%)
Female	365 (50.1%)
Age, years^b^	36.4 (23.8)
Town^a^	
Cizur Menor	483 (66.4%)
Astráin	41 (5.6%)
Gazólaz	21 (2.9%)
Larraya	20 (2.8%)
Muru Astráin	11 (1.5%)
Paternáin	59 (8.1%)
Sagüés	6 (0.8%)
Undiano	50 (6.9%)
Zariquiegui	37 (5.1%)
Body mass index^b^	22.4 (5.1)
BCG vaccine immunization status^aα^	
Not vaccinated	421 (57.8%)
Vaccinated	246 (33.8%)
Unknown	61 (8.4%)
Having received flu vaccine in this transmission season^aβ^	
No	573 (78.7%)
Yes	148 (20.3%)
Unknown	7 (1%)
Having had a flu or a cold in this transmission season^a^	
No	472 (64.8%)
Yes	240 (33%)
Unknown	15 (2.2%)
Comorbidities^a^*	
No	391 (53.7%)
Yes	338 (46.3%)
Reporting COVID-19 compatible symptoms since mid-February^a#^	
No	335 (46%)
Yes	393 (54%)
Close contact with COVID-19 confirmed or suspected case^a^	
No	469 (64.3%)
Yes	245 (33.6%)
Unknown	6 (4.7%)
Previously diagnosed with COVID-19^a^	
No	718 (98.6%)
Yes	10 (1.4%)
Visited a group gathering setting^a&^	
No	164 (22.5%)
Yes	564 (77.5%)
Household members^b^	4.3 (1.9)
Highest schooling level^a¥^	
Primary education	4 (0.9%)
Secondary education	104 (23.3%)
Technical studies	80 (17.9%)
University degree/Masters/PhD	250 (56.1%)
N/A	7 (1.7%)
Occupation class^a¥^	
Active worker	280 (62.8%)
Houseworker	18 (4%)
Permanent or temporary disability	1 (0.2%)
Retired	89 (20%)
Student	27 (6.1%)
Unemployed	7 (1.6%)
Other	1 (0.2%)
N/A	22 (4.9%)
Perceptions about information received from government and authorities^a$^	
I get enough useful information	146 (30.7%)
I get information but I still have some doubts	87 (18.3%)
I get information but I still have many doubts	99 (22.8%)
I don’t get enough useful information	135 (28.4%)
N/A	8 (1.7%)

^a^n/N (%) where N is the number of people in that age group (or overall, in the case of totals).

^b^Arithmetic Mean (SD).

*Comorbidities included diabetes, asthma, allergy, tuberculosis, Human Immunodeficiency Virus, Hepatitis A, B and C; depression, anxiety, dementia and other.

^#^Compatible symptoms included fever, chills, fatigue, myalgia, sore throat, anosmia, ageusia, cough, rhinorrhea, dyspnea, wheezing, chest pain, headache, abdominal pain, diarrhea, sputum from mid-February until date of interview.

^¥^Among participants aged 20 years or older.

^&^Exposures included visiting a sanitary center, grocery shop, bank, church, hairdresser, another city from mid-February until the end of March.

^$^Among participants aged 18 years or older.

^α^In Navarre BCG vaccine is not included in the immunization schedule since 1980.

^β^In Navarre flu vaccine is recommended to people over 60 years old.

Regarding socioeconomic characteristics, 0.9% among participants over 20 years of age had attended primary school as highest educational level, 23% secondary school, 18% technical studies and 56% had a university/masters or PhD degree (79.6%, 12.4% and 8% respectively). Sixty-three percent were active workers, 4% were homemakers, 0.2% declared permanently or temporary disability, 20% were retired, 6.1% were students and 1.6% were unemployed.

Finally, 30.7% perceived that they were receiving enough useful information by public health authorities and governments, 18.3% felt that they still had some doubts, 22.8% still had many doubts and 28.4% perceived not getting enough useful information.

### COVID-19 occupational and economic impact

We analysed the impact of the pandemic on the occupation and economy of the families (Supplementary Table 3). Among participants with 20 years of age or older, two participants with secondary education lost their job during the pandemic (1.9%), none because of reasons directly or indirectly related to the COVID-19 pandemic. Among the participants with technical education, four lost their job (5%) among which 3 (75%) were due to COVID-19 derived reasons; among those with superior university degrees, 5 (2%) lost their job, among which 2 (40%) were due to COVID-19. These differences did not reach statistical significance.

Twenty-five percent of participants with secondary education level reported being financially affected by the pandemic, the percentage rose to 36.3% of those with technical education and was lower in those with university degrees at 18.4% (46 out of 250). These differences were statistically significant (X2 = 11.045, *p*-value 0.011).

Among those with school level education, 1 out of 4 (25%) declared having struggles paying their expenses, 12/92 (13%) in the case of secondary education, 6 out of 75 (8%) in the case of technical education, and 13 out of 234 (5.7%) in the case of university degree level. Of these, 100%, 91.7%, 100% and 66.7%, respectively, declared that the difficulty was related or aggravated by the COVID-19 pandemic. These differences did not reach statistical significance. We found no significant differences in neither economic nor occupational impact between participant sex (data not shown).

### Behaviours during the first wave

Since mid-February 2020 to June 19th 2020, 43% of participants went to a health centre; 32.9% because of non-related COVID-19 symptoms, 7.2% for COVID-19-related symptoms, 23.5% accompanying someone else and 15.9% because of work.

Since mid-February until June 19th, adult participants (≥ 18 years old, n = 473) declared these exposures: 55.4% went to the supermarket, 29.9% went to a smaller grocery store, 9.1% went to the bank, 9.9% went to the church, 4% to the hairdresser, 24.3% went to Pamplona, 0.6% went to Madrid, 2.1% had been to another place in Spain and 0.4% (or 2 participants) had been to another place beyond Spain (Supplementary Table 4). Of those who went to the supermarket: 61.9% did not follow any preventive measure when bringing the groceries home, 33.7% cleaned them with soap, 20.1% with bleach, 2.6% put them in quarantine for one day, 4.8% for two or three days and 0.7% for four days or more.

A higher proportion of men (62.9%) went to the supermarket than women (47.9%) (X2 = 10.152, *p*-value < 0.001). Likewise, more men (29.1%) went to Pamplona—the nearest city—than women (19.5%) (X2 = 5.438, *p*-value 0.02), and to the bank—12.2% of men versus 6% of women—(X2 = 4.821, *p*-value 0.028).

### Seroprevalence

Fifty-six participants (7.7%) were seropositive for at least one of the isotype-antigen pairs tested (Table 2). Per age group, the seroprevalence was the lowest at 2.1% (95% CI 1.5–3) in 0–10 years old; the highest at 11.3% (95% CI 9.9–13) in 11–20 years; 7.9% (95% CI 6.7–9.2) in 21–50 years; 8.9% (95% CI 7.6–9.2) in 51–65 years; 7.5% (95% CI 6–9.1) in over 65 years. Seroprevalence in women was 8.52% (95% CI 6.1–11.8) and 7.4% (95% CI 5.6–10.7) in men. The estimated cumulative seroprevalence for the population was 7.9% (95% CI 7.3–8.6).

**Table 2:** Age-specific seroprevalences. Presence of IgM, IgA and IgG antibodies against S and/or RBD antigens was analyzed. The table presents the cumulative seroprevalence and seroprevalences for each isotype, their proportions and CI.

Isotype specificity	0–10 years of age (n = 142)	11–20 years of age (n = 159)	21–50 years of age (n = 177)	51–65 years of age (n = 157)	> 65 years of age (n = 93)	Estimated at total population (n = 728)
IgG and/or IgM and/or IgA	3/142 2.1% (1.5–3)	18/159 11.3% (9.9–13)	14/177 7.9% (6.7–9.2)	14/157 8.9% (7.6–9.2)	7/93 7.5% (6–9.3)	56/728 7.9% (7.3–8.6)
IgM	0	8/159 5.0% (4.1–6.2)	7/177 4.0% (3.1–5)	5/157 3.2% (2.4–4.2)	2/93 2.2% (1.4–3.3)	22/728 3.3% (2.9–3.8)
IgA	2/142 1.4% (0.9–2.2)	8/159 5.0% (4.7–7)	6/177 3.4% (2.6–4.3)	9/157 5.7% (4.7–7)	7/93 7.5% (6–9.4)	32/728 4.3% (3.8–4.8)
IgG	3/142 2.1% (1.5–3)	8/159 5.0% (4–6.2)	2/177 1.1% (0.7–1.7)	2/157 1.3% (0.8–2)	1/93 1.1% (0.6–2)	16/728 2.0% (1.7–2.4)

The estimated seroprevalence of IgG in the population was 2.0% (95% CI 1.7–2.4), 4.3% for IgA (95% CI 3.8–4.8), and 3.3% (95% CI 2.9–3.8) for IgM. Sixteen participants were only positive for IgG, 32 were only positive for IgA and 22 were only positive for IgM (Supplementary Fig. 1). Only two participants were seropositive for all three isotypes. Noteworthy, no participant with 0–10 years of age was found to be seropositive for IgM. Regarding antigen specificity, 17 were only positive for RBD, 27 only for S and 12 for both (at any of the three isotypes).

### Symptoms

Out of the 56 participants who were seropositive for any of the isotype-antigen pairs, 71.43% reported symptoms compatible with COVID-19 since mid-February *vs* 52.6% in seronegative participants. The most frequent reported symptoms among the seropositive were headache (35.7%), sore throat (35.7%), cough (32.1%) and rhinorrhoea (32.1%) (Supplementary Table 5). There were no differences in categories of symptoms reported across seropositive nor across seronegative between participant sex (data not shown).

Ten participants had been previously diagnosed with COVID-19, seven of them were diagnosed only based on symptoms and signs (during the first wave of the pandemic RT-qPCR testing was restricted to severe patients and/or high-risk groups), one via RT-qPCR and two via a serology test. All of them declared having had COVID-19 related symptoms. We did not detect antibodies in five previously diagnosed participants, however, four of them had only received a clinical diagnosis. The remaining had had a positive serology test.

**Figure 2:**
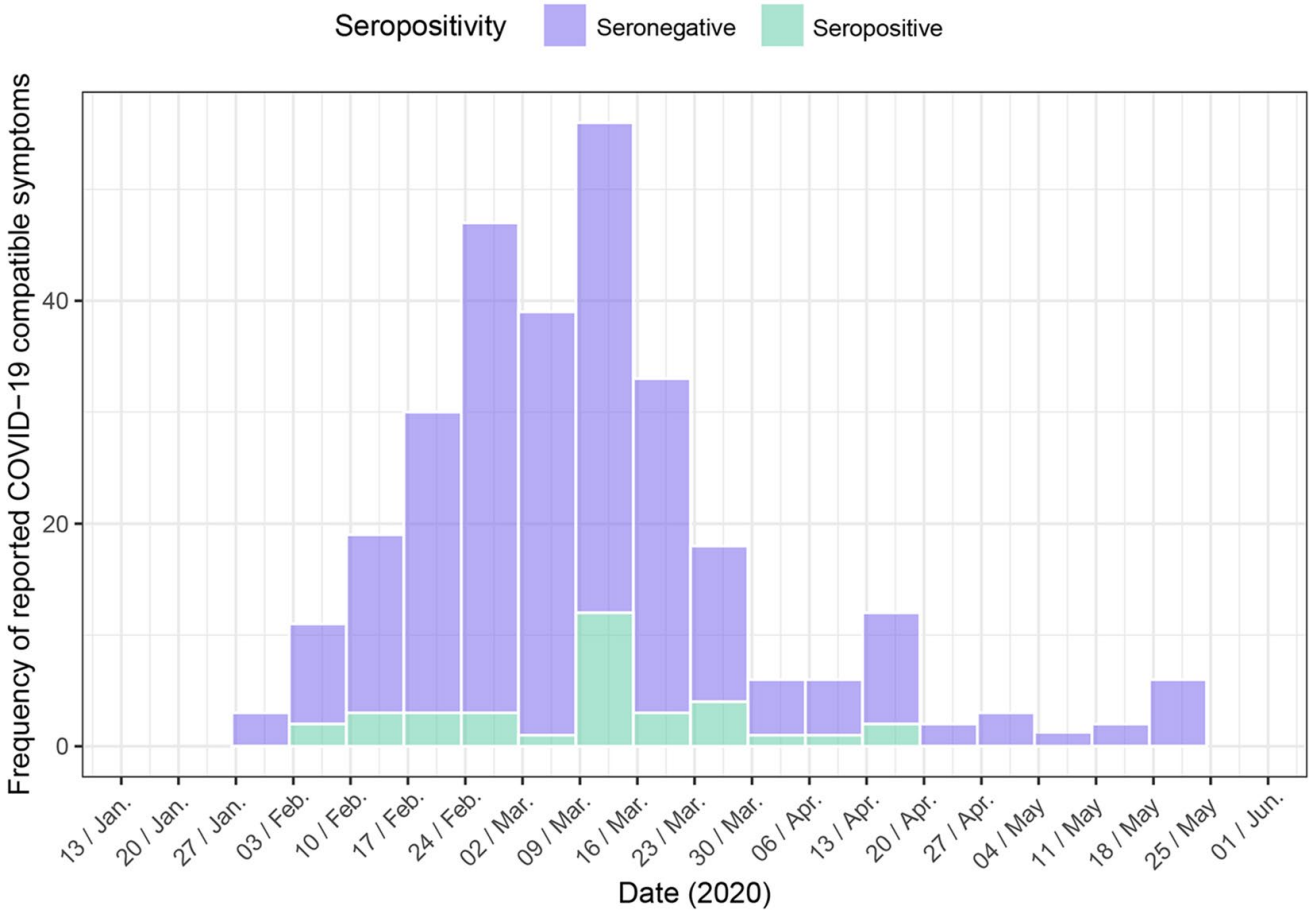
Frequency of dates of start of reported symptoms compatible with COVID-19 across time among participants. Purple bars represent seronegative participants and green bars seropositive participants.

Since mid-February until June, the highest frequency of onset of COVID-19 compatible symptoms was reported in the second week of March, right before the start of lockdown in Spain, and surprisingly in both seropositive and the seronegative participants (Fig. 2).

### Correlations between isotypes

**Figure 3:**
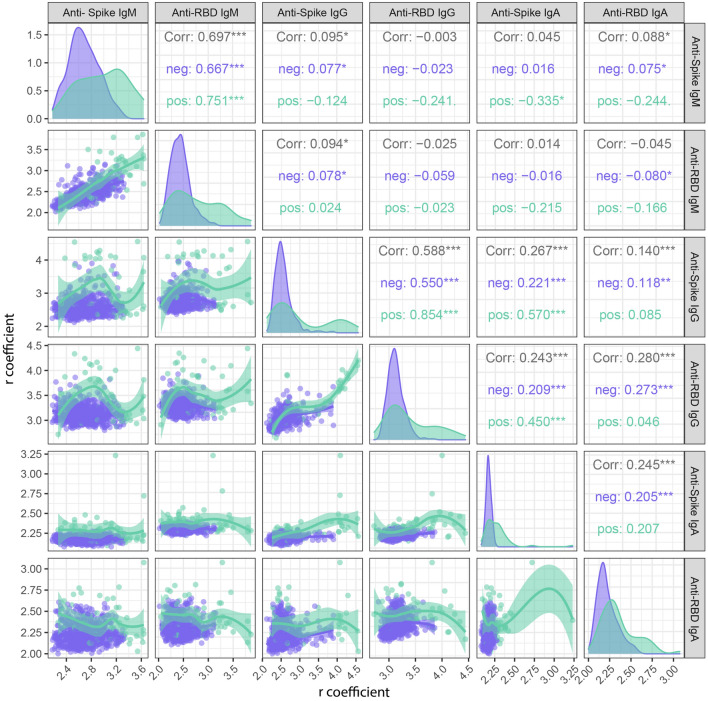
Scatter plots matrix representing correlations between isotype-antigen pairs. Two-sided spearman test was used to calculate the r_s_ correlation coefficients and *p*-values. Lines represent the fitted curves calculated using the loess method. Shaded areas represent 95% confidence intervals. “***” if the *p*-value is < 0.001, “**” if the *p*-value is < 0.01, “*” if the *p*-value is < 0.05. Purple dots, lines and shades represent seronegative participants and green seropositive ones.

We computed Spearman correlations between isotype-antigen pairs and found that, among the seropositive participants (n = 56), RBD-IgM levels highly correlated with S-IgM levels (r = 0.75), and S-IgG with RBD-IgG levels (r = 0.85) but this was not observed for IgA. Besides, S-IgA moderately correlated with S-IgG (r = 0.57) and with RBD-IgG (r = 0.45). IgM levels did not correlate with IgG nor IgA levels. Among the seronegative participants, IgGs and IgMs were found to correlate significantly (Fig. 3).

### Factors associated with seropositivity and antibody levels

Univariable analysis showed that adolescent age, having had flu symptoms or a cold in the previous transmission season, reporting COVID-19 compatible symptoms and having been diagnosed with COVID-19 were associated with higher odds of being seropositive (Table 3). When adjusting for one another in the multivariable analysis, results showed that the odds of being seropositive were lower in participants aged 0–10 than in all other groups and significantly lower than in adolescents (11–20; *p*-value < 0.05) and higher in those who had had COVID-19-like symptoms (Table 3).

**Table 3:** Univariable and multivariable analysis of factors associated with seropositivity (IgM and/or IgG and/or IgA).

Characteristics	Seropositive (n = 56)	Seronegative (n = 672)	Univariable analysis			Multivariable analysis (n = 712)		
			OR	(95% CI)	*p*	OR	(95% CI)	*p*
Gender^a^	56	672						
Female	31 (55.4%)	334 (49.7%)	1		0.23			
Male	25 (44.6%)	338 (50.3%)	0.70	(0.40; 1.25)				
Age, years^a^	56	672						
0–10	3 (5.4%)	139 (20.7%)	1		**0.065**	1		
11–20	18(32.1%)	141 (21%)	5.91	(1.80; 19.38)		5.03	(1.64; 21.94)	**0.012**
21–50	14 (25%)	163 (24.3%)	3.98	(1.20; 12.25)		3.24	(1.02; 14.39)	**0.071**
51–65	14 (25%)	143 (21.3%)	4.54	(1.27; 16.09)		3.34	(1.02; 14.97)	**0.068**
> 65	7 (12.5%)	86 (12.8%)	3.77	(0.95; 14.83)		3.89	(1.04; 18.55)	**0.056**
Town^a^	56	672						
Cizur Menor	38 (67.9%)	445 (66.2%)	1		0.507			
Atráin	3 (5.4%)	38 (5.6%)	0.88	(0.25; 3.09)				
Gazólaz	3 (5.4%)	18 (2.7%)	1.56	(0.44; 5.50)				
Larraya	0	20 (3%)	-					
Muru Astráin	1 (1.8%)	10 (1.5%)	0.95	(0.13; 6.92)				
Paternáin	4 (7.1%)	55 (8.2%)	1.10	(0.37; 3.33)				
Sagüés	2 (3.6%)	4 (0.6%)	5.76	(0.94; 35.34)				
Undiano	2 (3.6%)	48 (7.1%)	0.36	(0.08; 1.60)				
Zariquiegui	3 (5.4%)	34 (5.1%)	0.82	(0.17; 3.91)				
Body mass index ^b^	22 (4.3)	22.5 (5.2)	0.97	(0.92; 1.03)	0.73			
Blood group ^a^	24	219						
A	9 (37.6%)	130 (44.7%)	1		0.94			
AB	1 (4.1%)	6 (2.1%)	2.21	(0.32; 15.07)				
B	2 (8.3%)	21 (7.2%)	1.34	(0.31; 5.83)				
O	12 (50%)	134 (46%)	1.27	(0.55; 2.92)				
BCG vaccine immunization status^a^	49	617						
Not vaccinated	32 (65.3%)	388 (62.9%)	1		0.74			
Vaccinated	17 (34.7%)	229 (37.1%)	0.91	(0.51; 1.60)				
Having received flu vaccine in this transmission season ^a^	56	666						
No	45 (88.3%)	527 (79.1%)	1		0.46			
Yes	9 (16.7%)	139 (20.9%)	0.77	(0.39; 1.54)				
Having had the flu or a cold in this transmission season ^a^	55	611						
No	33 (60%)	446 (67.5%)	1		**0.015**	1		
Yes	22 (40%)	165 (32.5%)	1.89	(1.13; 3.15)		1.74	0.94–3.16	0.073
Comorbidities^a^*	56	672						
No	28 (50%)	309 (46%)	1		0.94			
Yes	28 (50%)	363 (54%)	0.98	(0.55; 1.75)				
Reporting COVID-19 compatible symptoms ^a#^	56	671						
No	16 (28.6%)	322 (48%)	1		**0.005**	1		
Yes	40 (71.4%)	349 (52%)	2.17	(1.24; 3.81)		1.99	1.07–3.10	**0.035**
Previously diagnosed with COVID-19 ^a^								
No	51 (91.1%)	665 (99.3%)	1		**0.0003**			
Yes	5 (8.93%)	5 (0.7%)	17.18	(3.74; 78.85)				
Having had a COVID-19 close contact								
No	33 (60%)	435 (66.2%)	1		0.744	1		
Yes, confirmed	16 (29%)	154 (23.4%)	1.37	(0.72;2.52)		1.25	0.64–2.37	0.493
Yes, suspected	6 (11%)	68 (10.4%)	1.16	(0.43–2.70)		1.06	0.38–2.53	0.902
Highest schooling level^a¥^								
University degree/Master/PhD	29 (19.8%)	383 (61.8%)	1		0.58			
Technical studies	10 (19.2%)	111 (17.9%)	1.17	(0.73; 2.33)				
Secondary education	13 (25%)	122 (19.7%)	1.37	(0.73; 2.55)				
Primary education	0 (0%)	4 (0.6%)	0					
Exposure to gatherings^a£^								
No	14 (25%)	203 (30.3%)	1		0.49			
Yes	42 (75%)	468 (69.7%)	1.28	(0.71; 2.29)				

^a^n/N (%) where N is the total number of participants who are seropositive or seronegative.

^b^Arithmetic Mean (SD).

^#^Compatible symptoms included fever, chills, fatigue, myalgia, sore throat, anosmia, ageusia, cough, rhinorrhea, dyspnea, wheezing, chest pain, headache, abdominal pain, diarrhea, sputum from mid-February until date of interview.

^¥^For participants over 20 years of age.

^£^Exposures included visiting a sanitary center, grocery shop, bank, church, hairdresser, another city from mid-February until the end of march.

Significant values are in bold.

To determine the factors associated with being seropositive for antigen and/or isotype specific when infected, antigen and isotype-specific multivariable models including only seropositive participants were performed. One-unit increase in BMI decreased by 0.82 (95% CI 0.68–0.96) the odds of being RBD seropositive (any isotype). Likewise, a unit increase in age was associated with an increase by 1.04 (95% CI 1.01–1.07) in the odds of being IgA seropositive. Finally, being male and a unit increase in age were associated with an increase by 4.83 (95% CI 1.22–22.91) and a decrease by 0.96 (95% CI 0.93–1.28) in the odds of being IgG seropositive, respectively (Supplementary Table 6).

None of the collected variables were uniquely associated with (i) IgG, (ii) IgM, (iii) IgA levels against (a) S, (b) RBD in univariable and multivariable linear regression models, in seropositive participants (for any isotype-antigen pair) only, except for levels of IgG against RBD which were 107% higher in male participants (95% CI 7.97–298.76). However, the models only included 36 observations which correspond to the seropositive participants for which we knew the date of onset of symptoms to be able to adjust the models for time since infection (Supplementary Tables 7–9).

## Discussion

We found an overall estimated seroprevalence of 7.9% (95% CI 7.3–8.6) in an age-stratified seroprevalence survey in a sample of 728 randomly selected, voluntarily registered inhabitants from a municipality of 3,925 inhabitants in Spain, conducted between June 12th and 19th 2020, during lockdown easing in Navarre. Participants showed a very heterogeneous immune response in terms of isotypes and antigens seropositivity, thus by analyzing IgA, IgM and IgG levels against S and RBD SARS-CoV-2 antigens we increased the sensitivity to detect seropositive responses. As expected, we found a higher seroprevalence than the proportion of diagnosed population (1.57%)^4^. ENE-COVID, a government large seroprevalence study performed in June 2020, estimated a seroprevalence of 5.2% (95% CI 4.9–5.5%) in the Autonomous Community of Navarre measured by a point-of-care rapid antigen test with 82.1% sensitivity and 100% specificity^26^, while our study estimated a seroprevalence of 7.9% (95% CI 1.7–2.4). The difference could be explained by the fact that we were assessing a larger panel of isotypes and antigens. When stratifying by age, taking into consideration all isotypes, we found a higher seroprevalence in aged 0–20 than ENE-COVID (7% vs 4.7%) and aged 21–50 (7.9% vs 5.6%); and a lower seroprevalence for aged 51–65 (8.9% vs 9.3%) and over 65 years (7.5% vs 8.2%)^4^, although the age distribution is consistent.

On the socioeconomic aspect, we have seen that four out of 728 participants lost their job for a pandemic-related reason, those with technical education were the most affected financially by the pandemic (36.25% of them) and those with school level education were the ones declaring having the most struggles playing their expenses (25% of them). Although our sample size was small and is extracted from a high-income town relative to its surroundings, these results are consistent with other reports predicting an aggravation of already existing inequalities across socioeconomic strata^27^. Oxfam estimated an increase in net income inequality of 1.0 points in 2020 in Navarre, measured in terms of the Gini index^28^.

Remarkably, we found that a higher proportion of men went to the supermarket, the bank, and the nearest city, than women. To our knowledge, this has not been reported scientifically, although informal communications corroborate it^29^. We hypothesize that this could be related to women tending to be more risk-averse than men^30^ or to women staying at home to take care of children, people with disabilities and elderly^31^.

We observed that most IgG seropositive participants were seronegative for IgA and vice versa. Recent publications show that IgG and IgA antibody levels are still stable at 12.5^32^, 13^33^, 16^14^ and up to 20.5 months after infection^34^, thus, seroreversion would not explain their seronegativity, but rather that the immune response profile mounted upon infection may be heterogeneous across individuals. The same variability was observed in epitope specificity: most participants had seropositive levels just for one of the two antigens assessed. In a previous paper, we had already shown this variability^35^. To our knowledge, this heterogeneity in antibody profiles has been seldom studied since results for each isotype-antigen pair are usually aggregated and not contrasted at individual level. Markmann et al. found that 65% of their seropositive participants had IgG antibodies against both RBD and N^36^. However, many studies show positive correlations between different isotype-antigen pair levels^36–38^, as we do. Anti-S and anti-RBD levels correlated for IgM and IgG, but not for IgA. Besides, S-IgA correlated with S-IgG and with RBD-IgG. This suggests that, albeit the correlations, panels assessing a wider range of antigens and evaluating both IgA and IgG might have higher sensitivity in determining seropositivity.

Chronologically, the week prior to lockdown witnessed the peak in the number of seropositive participants reporting onset of COVID-19 compatible symptoms. Assuming those seropositive individuals were experiencing COVID-19 at the time of COVID-19 compatible symptoms, it is surprising that the peak occurred before the start of lockdown. We would have expected the peak to occur one week after the start of lockdown (stopping the potential transmission chains), since the incubation period was around 6 days for the original SARS-CoV-2 variant^39^. Indeed, the peak of diagnosis in Navarre occurred the week of the 23rd to 29th March^4^. The small sample size could explain this shift as well as a recall bias, since the start of the state of alarm was used as a reference of time by fieldworkers. Furthermore, the frequency of seronegative participants declaring compatible symptoms peaked the same week. This leads us to reinforce the belief that some recall bias might have distorted the reported dates. Moreover, we hypothesize that—to some extent—a nocebo effect might partially explain the parallel frequency of symptoms in seropositive and seronegative participants. The nocebo effect—as opposed to the placebo effect—occurs when a preconceived belief or expectation leads to a negative effect, in this case developing or worsening of symptoms, and has been observed in severe COVID-19^40^ and in vaccination side-effects^41^.

We found that children between 0 and 10 years of age had less odds of being seropositive than those aged 11–20. A review reported this decreased risk of infection in children aged < 10 years^42^. Some observed that their adaptive immune response was less robust, and proposed that they might have benefited from a more efficient innate response^43–45^. Others have suggested that adults, because they have very likely been in contact with other coronaviruses, might be subjected to immune imprinting, where the immune system approaches the new virus as an exposure to an old one and hence mounts a less efficient response^46^. Besides, we also hypothesize that this might be explained by the differential measures that children underwent during the lockdown in Spain, as they were not allowed to leave their homes at all for almost a two-month period. Among the seropositive participants, we also found associations between higher age and higher odds of being specifically seropositive for IgA. While many studies are congruent with these findings^45,47,48^, other studies have found inverse association of age and antibody levels^49^.

Because of the heterogeneity in antigen-isotype responses across participants, we explored factors that might be associated with being seropositive for an antigen and/or isotype specifically once infected. We found an association between higher BMI and lower odds of RBD seropositivity for any isotype (0.82, 95% CI 0.68–0.96), which was described before by Frasca et al.^50^ and explained by the known influence of obesity in impairing the functioning of immune cells. Finally, being male was associated with higher odds of IgG seropositivity (4.83, 95% CI: 1.22–22.91) and higher levels of IgG-RBD. Male bias in COVID-19 disease severity has been widely reported and might be explained by differences in the immune response against the virus, as it has been described before for other viral infections, like influenza viruses, HIV or hepatitis viruses^51^. Klein et al. found higher antibody titers against S, S1 and RBD antigens in male^47^. Others have seen higher titers of neutralizing antibodies^36,48^ or higher levels of pro-inflammatory chemokines and cytokines^52^. However, some studies point towards a more robust response in females^52,53^.

When the outcomes were levels of a specific isotype-antigen pair, models did not provide any specific associated variable, except for levels of IgG against RBD which were 107% higher in male participants. However, we had a sample size of only 36 which probably reduces the statistical power of the model. A robust body of literature has associated severity with higher antibody levels^38,54^, unfortunately we did not collect degree of severity but only presence of symptoms.

The biggest limitation of the study is the low number of seropositive participants that restricts the power of regression models. Secondly, only 30.7% of the total population registered for the study and this may imply some self-selection bias and therefore would make the sample not fully representative of the population: people who had had symptoms were more likely to be interested, thus overestimating the seroprevalence, people with comorbidities might have had more interest to know their serostatus, while people already diagnosed where more likely to not register and, as a result, underestimate the true seroprevalence. Inhabitants over 65 years of age were under-represented in our sample and as a result, seroprevalence of this age group might be less representative of the population. In addition, we did not assess whether people had teleworked during lockdown, which should have been introduced in the regression models as a confounder. Finally, even if some degree of correlation is observed, levels of antibodies do not represent directly protection against infection and are just a piece of a complex immune response implicating many more actors.

Two years into the pandemic, 40% of the global population is believed to have been infected, 75% in Spain according to the IHME^55^. Knowledge has been created at an unprecedented speed, but key questions such as threshold for protection against infection, or duration of natural or acquired immunity against severe disease remain unknown. Seroprevalence studies like the one here presented helped in the early stages of the pandemic in determining the penetration of the infection at the population level to react accordingly. Now with the high vaccination coverage and lower rates of testing they shed light on the determinants and characteristics of the immune response to the vaccines and the virus, their duration and their efficacy against de variants of concern. Furthermore, household questionnaires inform on the socioeconomic aspects of the pandemic and when taken together with the serological data they elucidate populations that are more vulnerable to the infection and to its socioeconomic impact.

## Supplementary Information

Supplementary Information.

## Data Availability

The datasets generated during and/or analyzed during the current study are available in https://github.com/marta-ribes/SNAV.

## References

[1] WHO Coronavirus Disease (COVID-19) Dashboard | WHO Coronavirus Disease (COVID-19) Dashboard. https://covid19.who.int/.

[2] Wang H (2022). Estimating excess mortality due to the COVID-19 pandemic: A systematic analysis of COVID-19-related mortality, 2020. Lancet.

[3] Mukaigawara M (2022). An equitable roadmap for ending the COVID-19 pandemic. Nat. Med..

[4] Instituto de Salud Pública y Laboral de Navarra. *PRIMERA ONDA PANDÉMICA DE COVID-19 EN NAVARRA MARZO A JUNIO DE 2020*. (2020).

[5] Gobierno de Navarra. Plan y Normas para la Transición. https://gobiernoabierto.navarra.es/es/participacion/transitando-hacia-normalidad/plan-para-transicion (2020).

[6] Gobierno de España. ANEXO II.- PREVISIÓN ORIENTATIVA PARA EL LEVANTAMIENTO DE LAS LIMITACIONES DE ÁMBITO NACIONAL ESTABLECIDAS EN EL ESTADO DE ALARMA, EN FUNCIÓN DE LAS FASES DE TRANSICIÓN A UNA NUEVA NORMALIDAD. https://www.lamoncloa.gob.es/consejodeministros/resumenes/Documents/2020/28042020_Anexo%20II%20FASES.pdf (2020).

[7] Pinilla J (2021). The economic impact of the SARS-COV-2 (COVID-19) pandemic in Spain. Int. J. Environ. Res. Public Health.

[8] Bosch, N., Esteller-Moré, A. & Sorribas-Navarro, P. *El IEB Report 4/20 analiza el impacto de la COVID-19 en la pobreza: IEB*. https://ieb.ub.edu/el-ieb-report-4-20-analiza-el-impacto-de-la-covid-19-en-la-pobreza/(2020).

[9] Cheng MP (2020). Serodiagnostics for severe acute respiratory syndrome-related coronavirus-2. Ann. Intern. Med..

[10] Instituto de Salud Carlos III. Ministerio de Ciencia e Innovación. Gobierno de España. *ESTUDIO ENE-COVID: INFORME FINAL ESTUDIO NACIONAL DE SERO-EPIDEMIOLOGÍA DE LA INFECCIÓN POR SARS-COV-2 EN ESPAÑA*. http://www.thelancet.com/journals/lancet/article/PIIS0140-6736 (2020).

[11] Sette A, Crotty S (2022). Immunological memory to SARS-CoV-2 infection and COVID-19 vaccines. Immunol. Rev..

[12] Arkhipova-Jenkins I (2021). Antibody response after SARS-CoV-2 infection and implications for immunity a rapid living review background: The clinical significance of the antibody response. Ann. Intern. Med..

[13] Altawalah H, De Francesco MA (2021). Antibody responses to natural SARS-CoV-2 infection or after COVID-19 vaccination. Vaccines.

[14] Yang Y (2022). Longitudinal analysis of antibody dynamics in COVID-19 convalescents reveals neutralizing responses up to 16 months after infection. Nat. Microbiol..

[15] Ayuntamiento de la Cendea de Cizur. Datos de Interés. https://cendeadecizur.es/nuestra-cendea/datos-de-interes/.

[16] Infografias - Nastat - navarra.es. https://nastat.navarra.es/es/listado_infografias.

[17] Stadlbauer D (2020). SARS-CoV-2 seroconversion in humans: A detailed protocol for a serological assay, antigen production, and test setup. Curr. Protoc. Microbiol..

[18] Dobaño C (2021). Highly sensitive and specific multiplex antibody assays to quantify immunoglobulins M, A, and G against SARS-CoV-2 antigens. J. Clin. Microbiol..

[19] Santano R (2021). Agreement between commercially available ELISA and in-house Luminex SARS-CoV-2 antibody immunoassays. Sci. Reports.

[20] Jefatura del Estado. *LEY 14/2007, de 3 de julio, de Investigación biomédica*. 159 (BOE, 2007).

[21] Premkumar, L. *et al.* The RBD of the spike protein of SARS-group coronaviruses is a highly specific target of SARS-CoV-2 antibodies but not other pathogenic human and animal coronavirus Antibodies. *medRxiv Prepr. Serv. Heal. Sci.* 2020.05.06.20093377 (2020) 10.1101/2020.05.06.20093377.

[22] Wickham H (2019). Welcome to tidyverse. J. Open Source Softw..

[23] Wickham H (2016). ggplot2: Elegant Graphics for Data Analysis.

[24] Pebesma E (2018). Simple features for R: Standardized support for spatial vector data. R J..

[25] Lüdecke, D. sjPlot: Data Visualization for Statistics in Social Science. https://cran.r-project.org/web/packages/sjPlot/citation.html (2021).

[26] *Estudio Nacional de Sero-epidemiología de la Infección por SARS-CoV-2 en España*. (2020).

[27] Observatorio de la Realidad Social. *VI Informe sobre la pobreza y la desigualdad social en Navarra (2023)*. (2022).

[28] Oxfam Intermon. Informe Pobreza y Desigualdad. https://oxfam.app.box.com/s/90ijkmfp0tlfzd1q7hb64mka4q29xfvt (2020).

[29] ¿Quién se encarga de las tareas domésticas durante el confinamiento? Covid-19, mercado de trabajo y uso del tiempo en el hogar—Nada es Gratis. https://nadaesgratis.es/admin/quien-se-encarga-de-las-tareas-domesticas.

[30] Byrnes JP, Miller DC, Schafer WD (1999). Sex differences in risk taking: A meta-analysis. Psychol. Bull..

[31] Instituto Navarro para la Igualdad. *El Confinamiento En Primera Persona: Vivencias y Testimonios. El Impacto y las Consecuencias del Confinamiento en Mujeres de Navarra*. (2020).

[32] Dobaño C (2021). Persistence and baseline determinants of seropositivity and reinfection rates in health care workers up to 12.5 months after COVID-19. BMC Med..

[33] Gallais F (2021). Evolution of antibody responses up to 13 months after SARS-CoV-2 infection and risk of reinfection. EBioMedicine.

[34] Dobaño C (2022). Sustained seropositivity up to 20.5 months after COVID-19. BMC Med..

[35] Ortega N (2021). Seven-month kinetics of SARS-CoV-2 antibodies and role of pre-existing antibodies to human coronaviruses. Nat. Commun..

[36] Markmann AJ (2021). Sex disparities and neutralizing-antibody durability to SARS-CoV-2 infection in convalescent individuals. mSphere..

[37] Ma J (2020). COVID-19 patients in earlier stages exhaled millions of SARS-CoV-2 per hour. Clin. Infect. Dis..

[38] Wang H (2022). Clinical and antibody characteristics reveal diverse signatures of severe and non-severe SARS-CoV-2 patients. Infect. Dis. Poverty.

[39] Wassie GT, Azene AG, Bantie GM, Dessie G, Aragaw AM (2020). Incubation period of severe acute respiratory syndrome novel coronavirus 2 that causes coronavirus disease 2019: A systematic review and meta-analysis. Curr. Ther. Res. Clin. Exp..

[40] Daniali H, Flaten MA (2022). Experiencing COVID-19 symptoms without the disease: The role of nocebo in reporting of symptoms. Scand. J. Public Health.

[41] Sever PP (2022). Nocebo affects after COVID-19 vaccination. Lancet Reg. Heal. Eur..

[42] Irfan O, Li J, Tang K, Wang Z, Bhutta ZA (2021). Correspondence to: Risk of infection and transmission of SARS-CoV-2 among children and adolescents in households, communities and educational settings: A systematic review and meta-analysis. J Glob Health..

[43] Cohen CA (2021). SARS-CoV-2 specific T cell responses are lower in children and increase with age and time after infection. Nat. Commun..

[44] Pierce CA (2020). Immune responses to SARS-CoV-2 infection in hospitalized pediatric and adult patients. Sci. Transl. Med.

[45] Weisberg SP (2020). Distinct antibody responses to SARS-CoV-2 in children and adults across the COVID-19 clinical spectrum. Nat. Immunol..

[46] Stephenson, T. *et al.* Long COVID: The physical and mental health of children and non-hospitalised young people 3 months after SARS-CoV-2 infection; a national matched cohort study (The CLoCk) Study. *BMJ Open* **11**, (2021). 10.1136/bmjopen-2021-052838 PMC8392739 34446502

[47] Klein SL (2020). Sex, age, and hospitalization drive antibody responses in a COVID-19 convalescent plasma donor population. J. Clin. Invest..

[48] Mehew J, Johnson R, Roberts D, Harvala H (2020). Convalescent plasma for COVID-19: Male gender, older age and hospitalisation associated with high neutralising antibody levels, England, 22 April to 12 May 2020. Eurosurveillance.

[49] Yang HS (2021). Association of age with SARS-CoV-2 antibody response. JAMA Netw. Open.

[50] Frasca D (2021). Influence of obesity on serum levels of SARS-CoV-2-specific antibodies in COVID-19 patients. PLoS ONE.

[51] Jacobsen H, Klein SL (2021). Sex differences in immunity to viral infections. Front. Immunol..

[52] Takahashi T (2020). Sex differences in immune responses that underlie COVID-19 disease outcomes Overview of the study design. Nature.

[53] Schlickeiser S (2021). Disease severity, fever, age, and sex correlate with SARS-CoV-2 neutralizing antibody responses. Front. Immunol..

[54] Yan X (2022). Anti-SARS-CoV-2 IgG levels in relation to disease severity of COVID-19. J. Med. Virol..

[55] IHME. Estimation of total mortality due to COVID-19|Institute for Health Metrics and Evaluation. http://www.healthdata.org/special-analysis/estimation-excess-mortality-due-covid-19-and-scalars-reported-covid-19-deaths.

