# Supplementary material for: Seroprevalence and socioeconomic impact of the first SARS-CoV-2 infection wave in a small town in Navarre, Spain
Source: Sci Rep. 2023 Mar 8;13:3862. doi: 10.1038/s41598-023-30542-x (PMC9992915; doi:10.1038/s41598-023-30542-x)
Supplement: Supplementary file 1 — Supplementary Information. [file 41598_2023_30542_MOESM1_ESM.pdf]

Seroprevalence and Socioeconomic Impact of the First SARS-CoV-2 Infection Wave in a Small Town in Navarre, Spain

Supplementary Tables

Age groups	**0-10**	**11-20**	**21-50**	**51-65**	**>65**	**Total**
**Population censed**	536	755	1532	742	360	3,925
**Individuals registered**	210	264	358	289	97	1,218
**Sample size needed**	147	160	179	159	130	775
**Individuals recruited**	142	159	177	157	93	728

Supplementary Table 1. Per age group: population censed in Cendea de Cizur, individuals registered, sample size needed for the study and individuals recruited.

Characteristics	0-10 years of age (n=142)	11-20 years of age (n=159)	21-50 years of age (n=177)	51-65 years of age (n=157)	>65 years of age (n=93)
**Sex** [a]					
Male	63 (44.4%)	91 (57.2%)	85 (48%)	74 (47.1%)	50 (53.8%)
Female	79 (55.6%)	68 (42.8%)	92 (52%)	83 (52.9%)	43 (46.2%)
**Age, years** [b]	7.0 (2.7)	16.2 (2.8)	40.0 (8.6)	57.0(4.2)	74.3 (6.8)
**Town** [a]					
Cizur Menor	86 (60.6%)	127 (79.9%)	106 (59.9 %)	108 (68.8%)	56 (60.2%)
Astráin	4 (2.8%)	4 (2.5%)	13 (7.3%)	14 (8.9%)	6 (6.5%)
Gazólaz	2 (1.4%)	4 (2.5%)	3 (1.7%)	3 (1.9%)	9 (9.7%)
Larraya	1 (0.7%)	6 (3.8%)	6 (3.4%)	5 (3.2%)	2 (2.2%)
Muru Astráin	2 (1.4%)	3 (1.9%)	3 (1.7%)	2 (1.3%)	1 (1.1%)
Paternáin	20 (14.1%)	6 (3.8%)	21 (11.9%)	10 (6.4%)	2 (2.2%)
Sagüés	0	0	3 (1.7%)	2 (1.3%)	1 (1.1%)
Undiano	15 (10.6%)	7 (4.4%)	15 (8.5%)	7 (4.5%)	6 (6.5%)
Zariquiegui	12 (8.4%)	2 (1.3%)	7 (3.9%)	6 (3.8%)	10 (10.7%)
**Body mass index** [b]	16.4 (2.6)	20.5 (3.4)	23.9 (3.7)	25.5 (4.3)	26.8 (4.2)
**BCG vaccine immunization status** [a]					
Not vaccinated	133 (93.7%)	131 (82.4%)	107 (60.5%)	29 (18.5%)	21 (22.6%)
Vaccinated	5 (3.5%)	16 (10.1%)	48 (27.1%)	11 (71.3%)	65 (69.9%)
Unknown	4 (2.8%)	12 (7.5%)	22 (12.5%)	16 (10.2%)	7 (7.5%)
**Having received flu vaccine in this transmission season** [a]					
No	124 (87.3%)	148 (93.1%)	151 (85.3%)	118 (75.2%)	32 (34.4%)
Yes	17 (12%)	9 (5.7%)	24 (13.6%)	37 (23.6%)	61 (65.6%)
Unknown	1 (0.7%)	2 (1.3%)	2 (1.1%)	2 (1.3%)	0
**Having had a flu or a cold in this transmission season** [a]					
No	99 (69.7%)	96 (60.4%)	101 (57.1%)	107 (68.1%)	69 (74.2%)
Yes	41 (28.9%)	58 (36.5%)	74 (41.8%)	45 (28.7%)	22 (23.7%)
Unknown	2 (1.4%)	5 (3.1%)	2 (1.1%)	5 (3.2%)	2 (2.1%)
**Comorbidities** [a*]					
No	113 (79.6%)	89 (56%)	99 (55.9%)	68 (43.3%)	22 (23.7%)
Yes	29 (20.4%)	70 (44%)	78 (44.1%)	89 (56.7%)	71 (76.3%)
**Reporting COVID-19 compatible symptoms since mid-February** [a#]					
No	79 (55.6%)	59 (37.1%)	73 (41.2%)	68 (43.3%)	56 (60.2%)
Yes	63 (44.4%)	100 (62.9%)	104 (58.8%)	89 (56.7%)	37 (39.8%)
**Close contact with COVID19 confirmed or suspected case** [a]					
No	97 (68.3%)	98 (61.6%)	103 (58.2%)	96 (61.1%)	74 (79.6%)
Yes	38 (26.8%)	59 (37.1%)	73 (41.2%)	58 (36.9%)	17 (18.3%)
Unknown	0	0	1 (0.6%)	3 (2%)	2 (2.1%)
**Previously diagnosed with COVID19**[a]					
No	140 (98.6%)	155 (97.5%)	175 (98.9%)	155 (98.7%)	93 (100%)
Yes	2 (1.4%)	4 (2.5%)	2 (1.1%)	2 (1.3%)	0
**Visited a gathering setting** [a&]					
No	69 (48.6%)	52 (32.7%)	13 (7.3%)	8 (5.1%)	22 (23.7%)
Yes	73 (51.4%)	107 (67.3%)	164 (92.7%)	149 (94.9%)	71 (76.3%)
**Household members** [b]	5.2 (1.9)	5.3 (1.9)	4.3 (1.8)	3.7 (1.6)	2.7 (1.3)
**Highest schooling level** [a¥]					
Primary education	-	0 (0%)	0 (0%)	1 (0.7%)	3 (3.2%)
Secondary education	-	10 (55.5%)	28 (15.8%)	31 (19.7%)	35 (37.6%)
Technical studies	-	3 (16.7%)	32 (18.1%)	36 (22.9%)	9 (9.7%)
University degree /Masters /PhD	-	5 (26.8%)	117 (66.1%)	86 (54.8%)	42 (45.2%)
N/A	-	-	-	3 (1.9%)	4 (4.3%)
**Occupation class** [a¥]					
Active worker	.	6 (33.3%)	142 (80.2%)	123 (78.3%)	9 (9.7%)
Houseworker	.	0 (0%)	3 (1.7%)	10 (6.4%)	5 (5.4%)
Permanent or temporary disability	.	0 (0%)	1 (0.6%)	0	0
Retired	.	0 (0%)	0	17 (10.8%)	72 (77.4%)
Student	.	10 (55.6%)	17 (19.6%)	0	0
Unemployed	.	0 (0%)	5 (2.8%)	2 (1.3%)	0
Other	.	0 (0%)	1 (0.6%)	0	0
N/A	.	2 (11.1%)	8 (4.5%)	5 (3.2%)	7 (7.5%)
**Perceptions about information received from government and authorities** [a$]					
I get enough useful information	.	14 (29.17%)	57 (32.2%)	47 (29.9%)	28 (30.1%)
I get information but I still have some doubts	.	12 (25%)	32 (18.1%)	31 (19.7%)	12 (12.9%)
I get information but I still have many doubts	.	8 (16.7%)	38 (21.5%)	36 (22.9%)	17 (28.3%)
I don’t get enough useful information	.	12 (25%)	49 (27.7%)	42 (26.7%)	32 (34.4%)
N/A	.	2 (4.2%)	1 (0.6%)	1 (0.6%)	4 (4.3%)

Supplementary Table 2. Characteristics of study participants per age-group.

[a] n/N (%) where N is the number of people in that age group (or overall, in the case of totals)

[b] Arithmetic Mean (SD)

*Comorbidities included diabetes, asthma, allergy, tuberculosis, Human Immunodeficiency Virus, Hepatitis A, B and C; depression, anxiety, dementia and other.

[#]Compatible symptoms included fever, chills, fatigue, myalgia, sore throat, anosmia, ageusia, cough, rhinorrhea, dyspnea, wheezing, chest pain, headache, abdominal pain, diarrhea, sputum from mid-February until date of interview.

[¥]Among participants aged 20 years or older.

[&]Exposures included visiting a sanitary center, grocery shop, bank, church, hairdresser, another city from mid-February until the end of march.

[$]Among participants aged 18 years or older.

Characteristics	Primary education (n=4)	Secondary education (n=104)	Technical education (n=80)	University degree / Master / PhD (n=250)	Test statistic[c]	P-value
**Sex** [a]	4	104	80	250	X2=3.009	0.39
Female	3 (75%)	54 (51.9%)	34 (42.5%)	127 (50.8%)		
Male	1 (25%)	50 (48.1%)	46 (57.5%)	123 (49.2%)		
**Age, years** [b]	68.8 (10.1)	54.9 (20.1)	50.9 (13.3)	51.0 (14.1)	F=3.13	**0.026**
**Employment** [a]	4	101	80	245	X2=50.805	**0.001**
Housewife	0	0	2 (2.5%)	0		
Unemployed	0	26 (25.7%)	11 (13.8%)	27 (11%)		
Retired	3 (75%)	33 (32.7%)	11 (13.8%)	38 (15.5%)		
Employed	1 (25%)	42 (41.6%)	56 (70%)	180 (73.5%)		
**Lost employment because of COVID-19 pandemic (among those who became unemployed)** [a]	0	2	4	5	X2=3.135	0.09
Yes	0	0 (0%)	3 (75%)	2 (40%)		
No	0	2 (100%)	1 (25%)	3 (60%)		
**Financial affectation** [a]	4	104	80	250	X2=11.045	**0.011**
Yes	1 (25%)	26 (25%)	29 (36.3%)	46 (18.4%)		
No	4 (75%)	78 (75%)	51 (63.7%)	204 (81.6%)		
**Struggle to pay expenses** [a]	4	92	75	230	X2=6.487	0.09
Yes	1 (25%)	12 (13%)	6 (8%)	13 (5.7%)		
No	3 (75%)	80 (87%)	69 (92%)	217 (94.3%)		
**Due to COVID-19 pandemic** [a]	1	12	6	12	X2=4.511	0.211
Yes	1 (100%)	11 (91.7%)	6 (100%)	8 (66.7%)		
No	0 (0%)	1 (8.3%)	0 (0%)	4 (33.3%)		

Supplementary Table 3. Occupational and economic variables across educational level.

[a] n/N (%) where N is the total number of participants above 20 years of age or the subset who answered the question.

[b] Arithmetic Mean (SD)

[c] Joint F-test (ANOVA) is performed for numeric variables, and a Chi-square test of independence for categorical variables.

	Grouped		Females		Males			
Variable	N	%	N	%	N	%	Test statistic[c]	P-value
**Visited a sanitary centre**	476		236		240		X2=1.614	0.204
No	269	56.50%	126	53.40%	143	59.60%		
Yes	207	43.50%	110	46.60%	97	40.40%		
**Reason of visit**	207		110		97		X2=5.3	0.258
Accompanying someone	58	28%	29	26.4%	29	29.9%		
Other	32	15.50%	20	18.2%	12	12.4%		
Symptoms compatible with COVID-19	15	7.20%	7	6.4%	8	8.2%		
Symptoms incompatible with COVID-19	69	33.30%	32	29.1%	37	38.1%		
Work	33	15.90%	22	20%	11	11.3%		
**Visited:**								
Supermarket	473		236		237		X2=10.152	**0.001**
No	211	44.60%	123	52.10%	88	37.10%		
Yes	262	55.40%	113	47.90%	149	62.90%		
Grocery	472		233		239		X2=2.042	0.153
No	331	70.10%	171	73.40%	160	66.90%		
Yes	141	29.90%	62	26.60%	79	33.10%		
Bank	473		235		238		X2=4.821	**0.028**
No	430	90.90%	221	94%	209	87.80%		
Yes	43	9.10%	14	6%	29	12.20%		
Church	474		235		239		X2=3.18	0.075
No	427	90.10%	218	92.80%	209	87.40%		
Yes	47	9.90%	17	7.20%	30	12.60%		
Hairdresser	474		236		238		X2=0.237	0.626
No	455	96%	225	95.30%	230	96.60%		
Yes	19	4%	11	4.70%	8	3.40%		
Pamplona	473		236		237		X2=5.438	**0.02**
No	358	75.70%	190	80.50%	168	70.90%		
Yes	115	24.30%	46	19.50%	69	29.10%		
Madrid	475		236		239		X2=0	1
No	472	99.40%	235	99.60%	237	99.20%		
Yes	3	0.60%	1	0.40%	2	0.80%		
Another city in Spain	475		235		240		X2=0	1
No	465	97.90%	230	97.90%	235	97.90%		
Yes	10	2.10%	5	2.10%	5	2.10%		
Another city out of Spain	475		236		239		X2=0	0.406
No	473	99.60%	235	99.60%	238	99.60%		
Yes	2	0.40%	1	0.4%	1	0.4%		

Supplementary Table 4. Exposure to potential gatherings since the 14th of March until the 19th of June across sex. Joint F-test (ANOVA) is performed for numeric variables, and a Chi-square test of independence for categorical variables.

[a] n/N (%) where N is the total number of participants above 20 years of age or the subset who answered the question.

[b] Arithmetic Mean (SD)

[c] Joint F-test (ANOVA) is performed for numeric variables, and a Chi-square test of independence for categorical variables.

Symptom	Seropositive (n=56)	Seronegative (n=672)
Any symptom	71.4%	42.6%
Headache	35.7%	22.6%
Sore throat	35.7%	16.2%
Cough	32.1%	19.6%
Rhinorrhea	32.1%	16.4%
Fatigue	30.1%	15.6%
Diarrhea	19.6%	11.3%
Chills	19.6%	7.6%
Loss of taste	16.1%	1.3%
Fever over 37ºC	16%	7.9%
Productive cough	14.3%	8.3%
Loss of smell	12.5%	1.8%
Myalgia	8.9%	9.4%
Dyspnea	8.9%	3.9%
Wheeze	8.9%	4.5%
Chest pain	8.9%	4.9%
Abdominal pain	8.9%	5.8%
Nausea	7.1%	3.1%
Fever over 38ºC	7.1%	6.0%

Supplementary Table 5. Frequency of reported COVID-19 compatible symptoms since mid-February.

	UNIVARIABLE				MULTIVARIABLE			
	Beta (%)	Lower CI (%)	Upper CI (%)	*p*	OR	Lower CI	Upper CI	*p*
**RBD Seropositivity**								
Sex (male)	-93.29	-99.51	-19.80	0.04	0.37	0.1	1.25	0.112
Age	-0.26	-0.51	-0.03	0.04				
Fever (yes)	155.84	-81.64	3817.40	0.49				
Anosmia / Ageusia (yes)	-9.73	-96.42	2172.01	0.95				
Lower respiratory (yes)	-56.53	-96.37	400.25	0.50				
Upper respiratory (yes)	-62.91	-96.85	316.65	0.42				
Gastrointestinal (yes)	-19.70	-95.24	1250.79	0.88				
Body ache / fatigue (yes)	136.97	-79.54	2768.34	0.49				
Having had a cold in last winter season (yes)	-78.77	-98.39	157.93	0.23				
Smoker (yes)	-57.45	-99.17	1633.98	0.65				
BMI	-2.06	-3.71	-0.69	0.01	0.82	0.68	0.96	**0.019**
Allergy (yes)	1201.25	-45.21	53420.69	0.13	3.34	0.74	19.37	0.138
**IgA Seropositivity**								
Sex (male)	558.13	-45.07	8810.61	0.14	3.26	0.95	12.57	0.069
Age	0.25	0.01	0.51	0.05	1.04	1.01	1.07	**0.022**
Fever (yes)	567.08	-55.88	13002.04	0.18				
Anosmia / Ageusia (yes)	52.17	-93.71	4437.65	0.80				
Lower respiratory (yes)	-13.81	-92.73	916.27	0.91				
Upper respiratory (yes)	95.98	-82.96	2232.26	0.59				
Gastrointestinal (yes)	0.00	-93.99	1713.98	1.00				
Body ache / fatigue (yes)	-52.73	-96.11	458.92	0.55				
Having had a cold in last winter season (yes)	15.44	-90.71	1394.40	0.91				
Smoker (yes)	-96.26	-99.96	64.16	0.11	0.24	0.03	1.34	0.124
BMI	1.34	0.06	2.83	0.05				
Allergy (yes)	603.55	-70.48	28785.86	0.25				
**IgG Seropositivity**								
Sex (male)	2455.62	61.12	52956.23	0.03	4.83	1.22	22.91	**0.032**
Age	-0.35	-0.67	-0.08	0.02	0.96	0.93	1	**0.045**
Fever (yes)	1624.90	2.87	31314.59	0.05				
Anosmia / Ageusia (yes)	1244.20	-50.55	37850.95	0.12				
Lower respiratory (yes)	154.37	-82.73	3940.69	0.50				
Upper respiratory (yes)	-80.57	-98.88	187.17	0.24				
Gastrointestinal (yes)	650.70	-62.24	14408.56	0.18				
Body ache / fatigue (yes)	-50.50	-97.20	675.17	0.62				
Having had a cold in last winter season (yes)	-88.64	-99.55	96.17	0.15	0.29	0.05	1.28	0.124
Smoker (yes)	-6.46	-99.11	4201.49	0.97				
BMI	-0.01	-1.34	1.32	0.99				
Allergy (yes)	2623.23	14.63	73361.20	0.04	3.98	0.81	21.59	0.092

Supplementary Table 6. Univariable and multivariable analysis of factors associated with being seropositive for RBD antigen (regardless of isotype), IgA seropositive or IgG seropositive. Multivariable analysis included the variables with <0.2 p-value in the univariable models (n=56).

	UNIVARIABLE					MULTIVARIABLE				
	p		%	Lower CI %	Upper CI %	*p*		%	Lower CI %	Upper CI %
**IgM - RBD**										
Sex (male)	0.02	.	-55.72	-77.67	-12.18	0.07	ns	-48.13	-74.74	6.49
Days post symptom onset	0.13	ns	-1.26	-2.88	0.38	0.17	ns	-1.08	-2.63	0.49
Age	0.14	ns	-1.27	-2.95	0.44					
BMI	0.15	ns	-5.98	-13.67	2.40	0.36	ns	-3.79	-11.60	4.72
Body ache / fatigue (yes)	0.18	ns	64.34	-21.61	244.55					
Upper respiratory (yes)	0.39	ns	43.66	-38.39	234.95					
Allergy (yes)	0.54	ns	29.69	-44.63	203.77					
Fever (yes)	0.56	ns	-19.45	-61.65	69.18					
Smoker (yes)	0.58	ns	-27.32	-77.54	135.11					
Anosmia / Ageusia (yes)	0.68	ns	-15.67	-63.70	95.92					
Lower respiratory (yes)	0.81	ns	-8.73	-58.40	100.21					
Gastrointestinal (yes)	0.88	ns	-5.67	-57.01	107.01					
Having had a cold in last winter season (yes)	0.97	ns	-1.53	-53.13	106.89					
**IgM - S**										
Sex (male)	0.02	.	-49.5	-70.83	-12.66	0.08	ns	-40.2	-66.20	5.70
BMI	0.04	.	-6.89	-12.88	-0.49	0.12	ns	-5.19	-11.34	1.40
Age	0.10	ns	-1.15	-2.50	0.22					
Fever (yes)	0.18	ns	-32.66	-62.51	20.95					
Days post symptom onset	0.27	ns	-0.75	-2.08	0.61	0.33	ns	-0.61	-1.84	0.65
Anosmia / Ageusia (yes)	0.30	ns	-29.04	-63.58	38.25					
Allergy (yes)	0.33	ns	-28.42	-63.75	41.37					
Upper respiratory (yes)	0.34	ns	38.36	-29.98	173.40					
Smoker (yes)	0.71	ns	-16.20	-67.57	116.53					
Lower respiratory (yes)	0.74	ns	-9.76	-52.09	69.97					
Having had a cold in last winter season (yes)	0.79	ns	8.38	-40.41	97.12					
Body ache / fatigue (yes)	0.80	ns	8.05	-41.45	99.39					
Gastrointestinal (yes)	0.86	ns	5.61	-43.97	99.08					

Supplementary Table 7. *Variables associated with IgM levels (log10 transformed median fluorescence intensity (MFI) in univariable and multivariable regression models*. Multivariable analysis included the variables: (i) with <0.2 p-value in the univariable for the same Ig-Ag pair, (ii) with <0.2 p-value in the univariable for the same Ig, (iii) those that, according to literature, we have to account for (days post symptom onset) (n=36).

	UNIVARIABLE					MULTIVARIABLE				
	p		%	Lower CI %	Upper CI %	*p*		%	Lower CI %	Upper CI %
**IgA-RBD**										
Anosmia / Ageusia (yes)	0.03	.	35.51	2.33	79.46	0.06	ns	31.50	-1.36	75.30
Fever (yes)	0.15	ns	22.56	-7.56	62.49	0.15	ns	24.92	-8.35	70.27
Allergy (yes)	0.19	ns	23.89	-10.51	71.51	0.67	ns	7.47	-23.60	51.17
BMI	0.27	ns	1.86	-1.51	5.35					
Having had a cold in last winter season (yes)	0.35	ns	14.36	-14.06	52.17					
Gastrointestinal (yes)	0.38	ns	- 12.48	-35.36	18.49					
Days post symptom onset	0.51	ns	0.22	-0.44	0.88	0.90	ns	-0.04	-0.67	0.60
Upper respiratory (yes)	0.57	ns	9.78	-21.25	53.02					
Lower respiratory (yes)	0.71	ns	-5.42	-30.35	28.43					
Age	0.73	ns	0.12	-0.57	0.81					
Body ache / fatigue (yes)	0.79	ns	-3.76	-28.43	29.41					
Smoker (yes)	0.82	ns	5.26	-33.51	66.65					
Sex (male)	0.98	ns	0.33	-24.86	33.94					
**IgA-S**										
Fever (yes)	0.03	.	36.33	3.19	80.12	0.12	ns	30.72	-7.50	84.73
Allergy (yes)	0.03	.	41.77	2.89	95.33	0.34	ns	20.06	-17.98	75.75
Anosmia / Ageusia (yes)	0.10	ns	30.56	-5.42	80.23	0.22	ns	21.52	-11.86	67.52
Lower respiratory (yes)	0.14	ns	- 20.32	-41.26	8.07					
Upper respiratory (yes)	0.19	ns	- 19.78	-42.56	12.05					
Having had a cold in last winter season (yes)	0.22	ns	- 16.35	-37.45	11.89					
Smoker (yes)	0.36	ns	- 19.20	-49.32	28.83					
BMI	0.41	ns	1.44	-2.04	5.04					
Sex (male)	0.54	ns	9.34	-18.61	46.90					
Age	0.55	ns	-0.21	**-0.91**	0.50					
Days post symptom onset	0.82	ns	-0.08	-0.76	0.61	0.34	ns	-0.33	-1.04	0.37
Gastrointestinal (yes)	0.83	ns	-3.39	-29.47	32.34					
Body ache / fatigue (yes)	0.99	ns	0.11	-26.17	35.75					

Supplementary Table 8. Variables associated with IgA levels (log10 transformed median fluorescence intensity (MFI) in univariable and multivariable regression models. Multivariable analysis included the variables were: (i) the ones with <0.2 p-value in the univariable for the same Ig-Ag pair, (ii) the ones with <0.2 p-value in the univariable for the same Ig, (iii) those that, according to literature, we have to account for (days post symptom onset) (n=36).

	UNIVARIABLE					MULTIVARIABLE				
	p		%	Lower CI %	Upper CI %	*p*		%	Lower CI %	Upper CI %
**IgG - RBD**										
Allergy (yes)	0.01	*	198.51	38.28	544.38	0.06	ns	121.23	-4.62	413.41
Having had a cold in last winter season (yes)	0.01	*	-61.18	-80.09	-24.30	0.12	ns	-39.61	-68.51	15.79
Upper respiratory (yes)	0.01	.	-63.93	-83.49	-21.18	0.07	ns	-49.04	-75.35	5.34
Sex (male)	0.01	.	137.68	20.45	369.02	0.03	.	107.55	7.97	298.96
Fever (yes)	0.04	.	107.00	2.38	318.53	0.73	ns	13.82	-46.51	142.20
Age	0.05	.	-1.70	-3.33	-0.03	0.16	ns	-1.00	-2.40	0.42
Gastrointestinal (yes)	0.41	ns	37.96	-36.81	201.19					
Body ache / fatigue (yes)	0.42	ns	-26.12	-65.29	57.24					
BMI	0.45	ns	3.37	-5.29	12.83					
Lower respiratory (yes)	0.47	ns	32.12	-39.61	189.02					
Anosmia / Ageusia (yes)	0.66	ns	20.43	-48.12	179.58	0.21	ns	-35.10	-67.61	30.05
Days post symptom onset	0.74	ns	0.28	-1.42	2.01	0.94	ns	-0.05	-1.56	1.47
Smoker (yes)	0.80	ns	-14.10	-73.66	180.09					
**IgG - S**										
Sex (male)	0.02	.	311.22	24.34	1260.00	0.11	ns	191.09	-23.35	1005.41
Allergy (yes)	0.03	.	374.17	17.89	1807.15	0.21	ns	190.21	-47.92	1517.05
Age	0.05	ns	-2.84	-5.65	0.05	0.19	ns	-1.88	-4.69	1.02
Anosmia / Ageusia (yes)	0.06	ns	285.74	-3.69	1444.90	0.44	ns	71.38	-58.54	608.38
Having had a cold in last winter season (yes)	0.06	ns	-68.92	-90.92	6.32	0.53	ns	-33.56	-82.41	151.04
Upper respiratory (yes)	0.07	ns	-73.26	-93.54	10.77	0.42	ns	-44.47	-87.39	144.59
Fever (yes)	0.15	ns	150.52	-29.06	784.72	0.86	ns	14.66	-75.47	435.87
Body ache / fatigue (yes)	0.22	ns	-55.21	-87.76	63.83					
BMI	0.25	ns	9.08	-6.17	26.80					
Gastrointestinal (yes)	0.41	ns	74.24	-55.21	577.80					
Days post symptom onset	0.74	ns	-0.49	-3.41	2.52	0.58	ns	-0.84	-3.85	2.28
Smoker (yes)	0.88	ns	16.22	-85.15	809.58					
Lower respiratory (yes)	0.89	ns	-8.83	-76.87	259.40					

Supplementary Table 9. *Variables associated with IgG levels (log10 transformed median fluorescence intensity (MFI) in univariable and multivariable regression models.* Multivariable analysis included the variables were: (i) the ones with <0.2 p-value in the univariable for the same Ig-Ag pair, (ii) the ones with <0.2 p-value in the univariable for the same Ig, (iii) those that, according to literature, we have to account for (days post symptom onset) (n=36).

COMORBIDITY	n	% over total participants
Allergy	135	18.5 %
Hypertension	79	10.8 %
Asthma	47	6.4 %
Anxiety	27	3.7 %
Diabetes	23	3.2 %
Depression	16	2.2 %
Rhinitis/sinusitis	6	0.8 %
Hyperactivity Disorder	5	0.7 %
Dementia	2	0.3 %
Hepatitis	2	0.3 %
Guillaume-Barré Syndrome	1	0.1 %
Raynaud Syndrome	1	0.1 %
Gilbert Syndrome	1	0.1 %
Psoriasis	1	0.1 %
TB	0	0.0 %
HIV	0	0.0 %

Supplementary Table 10. Comorbidities declared by study participants.

Supplementary Figures

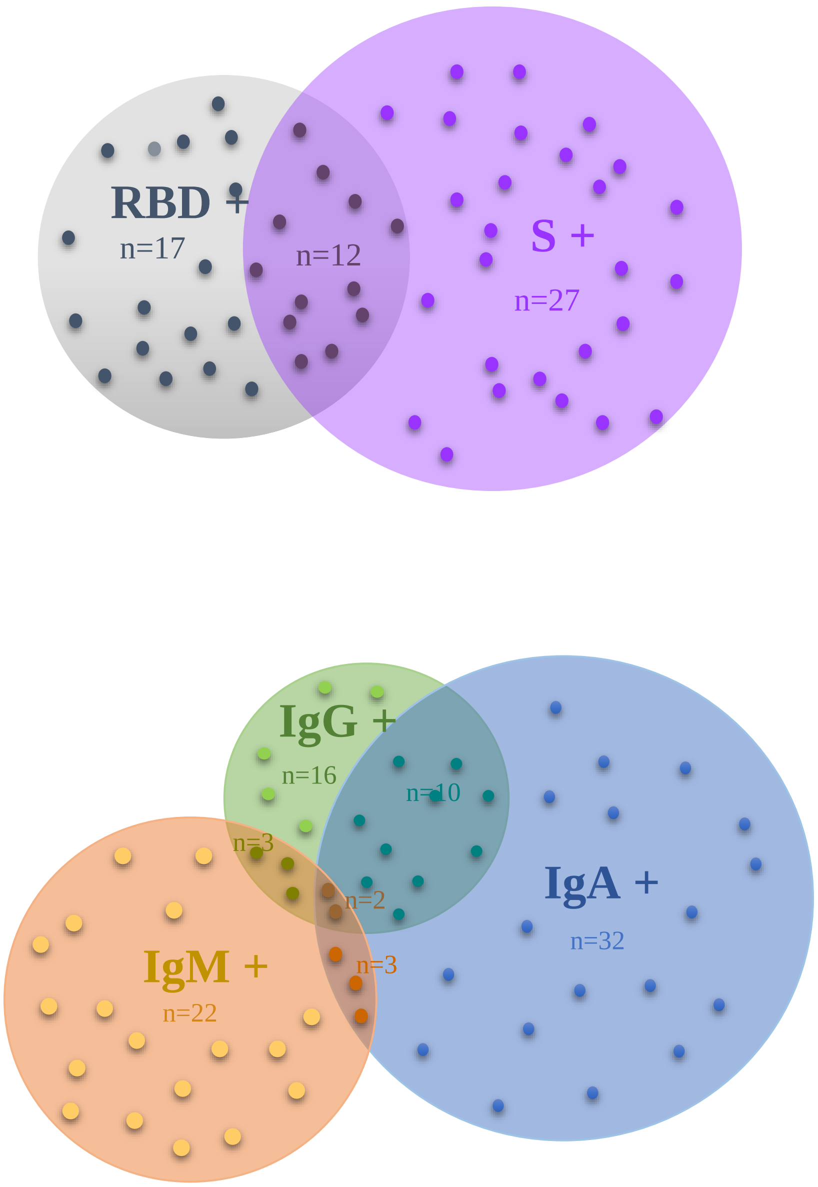

Supplementary Figure 1. Venn Diagrams of the number of participants seropositive for each antigen and for their combinations and for each isotype and their combinations.
